# The Milk Thistle (*Silybum marianum*) Compound Silibinin Inhibits Cardiomyogenesis of Embryonic Stem Cells by Interfering with Angiotensin II Signaling

**DOI:** 10.1155/2018/9215792

**Published:** 2018-12-13

**Authors:** Enas Hussein Ali, Fatemeh Sharifpanah, Amer Taha, Suk Ying Tsang, Maria Wartenberg, Heinrich Sauer

**Affiliations:** ^1^Department of Physiology, Faculty of Medicine, Justus Liebig University Gießen, Germany; ^2^School of Life Sciences, The Chinese University of Hong Kong, Hong Kong; ^3^Key Laboratory for Regenerative Medicine, Ministry of Education, The Chinese University of Hong Kong, Hong Kong; ^4^Department of Internal Medicine I, Division of Cardiology, Angiology, Pneumology, and Intensive Medical Care, University Hospital Jena, Friedrich Schiller University Jena, Germany

## Abstract

The milk thistle (*Silybum marianum* (L.) Gaertn.) compound silibinin may be an inhibitor of the angiotensin II type 1 (AT_1_) receptor which is expressed in differentiating embryonic stem (ES) cells and is involved in the regulation of cardiomyogenesis. In the present study, it was demonstrated that silibinin treatment decreased the number of spontaneously contracting cardiac foci and cardiac cell areas differentiated from ES cells as well as contraction frequency and frequency of calcium (Ca^2+^) spiking. In contrast, angiotensin II (Ang II) treatment stimulated cardiomyogenesis as well as contraction and Ca^2+^ spiking frequency, which were abolished in the presence of silibinin. Intracellular Ca^2+^ transients elicited by Ang II in rat smooth muscle cells were not impaired upon silibinin treatment, excluding the possibility that the compound acted on the AT_1_ receptor. Ang II treatment activated extracellular signal-regulated kinase 1/2 (ERK1/2), c-Jun NH_2_-terminal kinase (JNK), and p38 mitogen-activated protein kinase (MAPK) pathways in embryoid bodies which were abolished upon silibinin pretreatment. In summary, our data suggest that silibinin inhibits cardiomyogenesis of ES cells by interfering with Ang II signaling downstream of the AT_1_ receptor.

## 1. Introduction

Silibinin is the pharmacologically most important compound of silymarin which contains different flavonolignans and is an extract from milk thistle (*Silybum marianum* (L.) Gaertn., Asteraceae) [1]. The pharmacologic actions of silibinin have been mainly attributed to its hepatoprotective and anticancer properties [2]. However, silibinin has been also shown to be pharmacologically active in the cardiovascular system. In this respect, it has been demonstrated to exert cardioprotective properties, e.g., following isoproterenol-induced cardiac myocyte injury [3, 4] or doxorubinin-mediated cardiotoxicity [5]. Moreover, silibinin reduced blood pressure and the incidence of postocclusion arrhythmias in spontaneously hypertensive rats, and it was suggested that this compound may be beneficial when used in hypertensive patients who develop acute myocardial infarction [6]. Silymarin exhibited significant antihypertensive activity in a DOCA salt model of hypertension [7]. In anesthetized open chest cats, silibinin lowered the amplitude and duration of diastolic blood pressure and produced a marked depression of cardiac contractility [8], suggesting that silibinin affects the hemodynamic properties of the heart.

The mechanism by which silibinin is pharmacologically active in the heart is so far not known. Recently, it was suggested that silibinin may act as an antagonist of angiotensin receptor 1 (AT_1_) since it inhibited Ang II-mediated Ca^2+^ signals in Chinese hamster ovary (CHO) cells overexpressing the AT_1_ receptor [9]. The physiological impact of Ang II in the adult heart is so far not sufficiently investigated. Cardiomyocytes express the AT_1_ as well as the AT_2_ receptor [10]. In cultured cardiomyocytes, AT_1_ receptors have been demonstrated to mediate apoptosis [11] or to promote hypertrophy [12, 13], depending on the experimental conditions and the expression pattern of AT receptor subtypes.

The renin-angiotensin aldosteron system (RAAS) is likely crucial for proper embryogenesis. Components of the RAAS are highly expressed in many tissues during embryonic development. AT_1_ receptor expression is downregulated shortly after birth, whereas the AT_2_ receptor is upregulated, suggesting a potential role of AT_1_ in cell/tissue differentiation processes during embryogenesis and a potential role of AT_2_ in adult organ function [14]. In fetal ovine cardiomyocytes, Ang II stimulates hyperplastic growth [15], indicating that Ang II is involved in fetal heart growth. In ES cells, Ang II has been shown to regulate glucose uptake [16], supporting the notion that Ang II may play a role in energy metabolism during embryogenesis. Notably, Ang II has been demonstrated to stimulate cardiomyogenesis [17] and smooth muscle differentiation [18] of ES cells. In differentiating ES cell-derived embryoid bodies, the AT_1_ receptor is expressed already at very early stages of cardiac cell commitment. Moreover, besides insulin-like growth factor (IGF) receptors, AT_1_ receptor expression has been shown to be present in human cardiac stem cells [19], thus outlining an impact of Ang II signaling in differentiation and/or cardiac progenitor cell proliferation.

In the present study, we investigated the effect of silibinin on cardiomyogenesis of ES cells. Our data demonstrate that silibinin inhibited cardiac cell differentiation and contraction frequency. Notably, silibinin abolished Ang II-mediated procardiogenic effects and decreased Ca^2+^ spiking frequency without interfering with Ang II receptor function. In conclusion, our data suggest that silibinin interferes with Ang II-mediated signaling pathways by inhibition of mitogen-activated protein kinases (MAPKs) downstream of the AT_1_ receptor.

## 2. Materials and Methods

### 2.1. Materials

Silibinin-C-2′,3-dihydrogen succinate, disodium salt (Legalon SIL) was a generous gift from MEDA Pharma GmbH & Co. KG (Bad Homburg, Germany). Drug substance was as follows: silibinin-C-2′3-dihydrogen succinate, 528.5 mg (corresponding to 476 mg mono-, dihydrogensuccinate sodium salts (HPLC)) equivalent to 350 mg of silibinin. The drug substance contained 70 mg inulin (USP) as excipient. Ang II, FGF-2, L-NAME, and LY294002 were purchased from Sigma-Aldrich (Munich, Germany). Eicosapentanoic acid (EPA) was from Tocris Bioscience (Wiesbaden, Germany).

### 2.2. Cell Culture of ES Cells and Embryoid Body Formation

Mouse ES cells (line CCE) were grown on mitotically inactivated feeder layers of primary mouse embryonic fibroblasts (purchased from Amsbio, Abingdon, UK) in Iscove's basal medium (Biochrom, Berlin, Germany) supplemented with 15% heat-inactivated (56°C, 30 min) foetal calf serum (FCS) (Sigma-Aldrich), 2 mM glutamine, (PAA, Cölbe, Germany), 100 *μ*M 2-mercaptoethanol (Sigma-Aldrich), 1% (v/v) NEA nonessential amino acid stock solution (Biochrom), 1 mM Na^+^-pyruvate (Biochrom), 0.4% penicillin/streptomycin (Biochrom), and 1000 U/ml leukemia inhibitory factor (LIF) (Merck Millipore, Darmstadt, Germany) in a humidified environment containing 5% CO_2_ at 37°C, and passaged every 2-3 days. Adherent cells were enzymatically dissociated using 0.05% trypsin-EDTA in phosphate-buffered saline (PBS) (Thermo Fisher Scientific, Waltham, MA, USA) and seeded at a density of 3 · 10^6^ cells/ml in 250 ml siliconized spinner flasks (CellSpin, Integra Biosciences, Fernwald, Germany) containing 125 ml Iscove's medium supplemented as described above, but devoid of LIF. Following 24 h, 125 ml medium was added to give a final volume of 250 ml. The spinner flask medium was stirred at 20 r.p.m. using a stirrer system (Integra Biosciences). The spinning direction was changed every 1440°. 125 ml cell culture medium was exchanged every day.

### 2.3. Immunohistochemistry

As the primary antibody, a mouse monoclonal anti-*α*-actinin antibody (Abcam, Cambridge, UK) (dilution 1 : 100) was used. The embryoid bodies were fixed in ice-cold methanol for 20 min at −20°C and washed with phosphate-buffered saline (PBS) containing 0.01% Triton X-100 (PBST). Blocking against unspecific binding was performed for 60 min at room temperature with 10% heat-inactivated FCS (AppliChem, Darmstadt, Germany) dissolved in PBST. Embryoid bodies were subsequently incubated overnight at 4°C with primary antibody (dilution 1 : 100) dissolved in PBST supplemented with 10% FCS. The embryoid bodies were thereafter washed three times with PBST and reincubated for 1 h at room temperature in the dark with Alexa Fluor 647 sheep anti-mouse IgG secondary antibody (Jackson ImmunoResearch Laboratories, West Grove, PA, USA) diluted 1 : 100 in PBST containing 10% FCS. After washing three times with PBST, the tissues were stored in PBST until inspection.

### 2.4. Western Blot Analysis

Protein extraction was carried out after washing the embryoid bodies in PBS and lysing in RIPA lysis buffer (50 mM Tris-HCl (pH 7.5), 150 mM NaCl, 1 mM EDTA (pH 8.0), 1 mM glycerophosphate, 0.1% SDS, and 1% Nonidet P-40) supplemented with protease inhibitor cocktail (PXBioVisioN, Hannover, Germany) and phosphatase inhibitor cocktail (Sigma-Aldrich) for 20 min on ice. Samples were centrifuged at 24,700*g* for 10 min at 4°C to pellet the debris. After determination of the protein concentration using a Lowry protein assay, 20 *μ*g of protein samples was boiled for 10 min at 70°C, separated in PAGE Ex Precast gels (4–12%) (Lonza, Cologne, Germany), and transferred to PVDF membranes by the XCell SureLock Mini-Cell Blot Module (Invitrogen) at 180 mA for 90 min. Membranes were blocked with 5% (wt/vol) dry fat-free milk powder in Tris-buffered saline with 0.1% Tween (TBST) for 60 min at room temperature. Incubation with the primary antibody was performed at 4°C overnight. Used primary antibodies were phospho-ERK (Thr202/Tyr204), phospho-38 (Thr180/Tyr182), phospho-JNK (Thr183/Tyr185), and cleaved caspase 3 (Asp 175) (Cell Signaling Technology Europe, Frankfurt, Germany). Primary antibodies against either vinculin or *β*-actin (Sigma-Aldrich), which are housekeeping proteins, were used for the standardization of blotting. After washing with 0.1% TBST, the membrane was incubated with a horseradish peroxidase- (HRP-) conjugated secondary antibody (dilution 1 : 1000) (Abcam, Cambridge, UK) for 60 min at room temperature. The blot was developed using an enhanced chemiluminescence (ECL) solution to produce a chemiluminescence signal. For quantification, the density of the protein bands on the western blot image, which was acquired using the Peqlab gel documentation system (VWR International, Darmstadt, Germany), was assessed by ImageJ [20]. The final quantification reflects the relative amounts of protein as a ratio of each target protein band to the respective housekeeping protein.

### 2.5. Recording of Intracellular Ca^2+^ Concentrations

Intracellular Ca^2+^ was recorded in single cardiac contracting cells. Single cell preparations were obtained by enzymatic digestion of 7-day-old embryoid bodies for 30 min at 37°C in PBS containing 2 mg/ml Collagenase B (Roche, Mannheim, Germany). Dissociated single cells were plated onto gelatin-coated cover slips in 24-well cell culture plates (Greiner Bio-One GmbH, Frickenhausen, Germany), and cultivated in Iscove's medium supplemented with 15% FCS. Following 24 h of culture, cells were loaded in serum-free medium with 1 *μ*M Fluo-4/AM (Life Technologies) for 30 min. Subsequently, the cover slips were transferred in fresh serum-free cell culture medium to the incubation chamber of a confocal laser scanning microscope (Leica SP2, AOBS, Leica, Bensheim, Germany). Fluorescence excitation was performed at 488 nm, and emission was recorded at 500–550 nm. Sampling rate was 2 frames/s. The fluorescence emission of single cells was assessed by using the image analysis software of the confocal setup.

### 2.6. Statistical Analysis

For statistical analysis, GraphPad InStat statistics software (GraphPad Software Inc., La Jolla, CA) was used. Data are given as mean values ± standard deviation (S.D.), with *n* denoting the number of experiments performed with independent ES cell cultures. In each experiment, at least 20 culture objects were analyzed unless otherwise indicated. Student's *t*-test for unpaired data and one-way ANOVA was applied as appropriate for statistical analysis. A value of *P* ≤ 0.05 was considered significant.

## 3. Results

### 3.1. Inhibition of Cardiomyogenesis of ES Cells and Contractility of Differentiated Cardiac Cells

**Figure 1:**
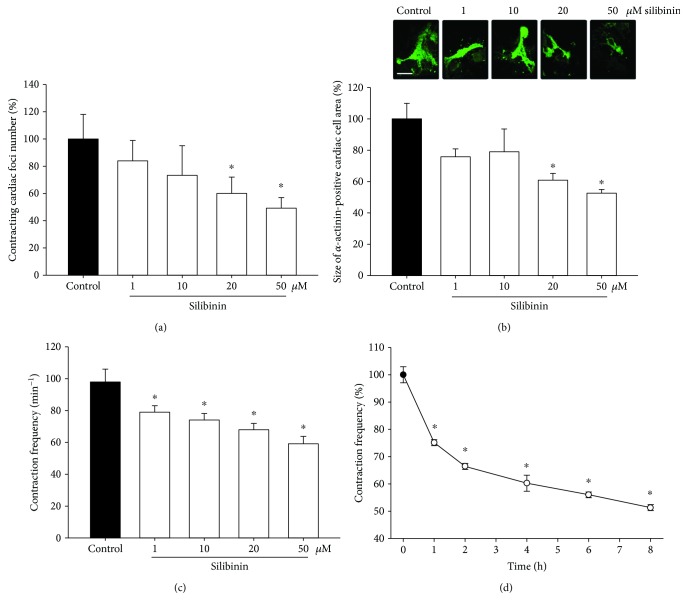
Effect of increasing doses of silibinin on (a) the number of contracting cardiac foci differentiated from ES cells (*n* = 4), (b) the size of cardiac foci (*n* = 3), and (c) the contraction frequency (*n* = 4). (d) Image shows the decay in contraction frequency over time following treatment with 20 *μ*M silibinin (*n* = 3). The images in (b) show representative cardiac cell areas immunolabeled with an antibody against *α*-actinin. The bar represents 300 *μ*m. ^∗^ *P* ≤ 0.05, significantly different to the untreated control.

To examine the effects of silibinin on the differentiation of cardiomyocytes, embryoid bodies were treated from day 3 until day 10 with different concentrations of silibinin (1 *μ*M, 10 *μ*M, 20 *μ*M, and 50 *μ*M). From day 7 to day 10, the number of contracting cardiac foci, the size of the *α*-actinin-positive cardiac area, and the contraction frequency were assessed. It was observed that silibinin treatment dose-dependently decreased the number of contracting foci (Figure 1(a)), the size of *α*-actinin-positive cell areas (Figure 1(b)), and the contraction frequency (Figure 1(c)). To investigate in more detail the time course of decline in the contraction frequency of cardiac areas, 10-day-old embryoid bodies were incubated with 20 *μ*M silibinin and the decrease in contraction frequency was assessed over time. It was observed that within 1 h of incubation a significant slowdown of contraction frequency occurred, which decreased further over 8 h (Figure 1(d)). To exclude the possibility that silibinin induced apoptosis in embryoid bodies, cleaved caspase-3 was assessed after 7 days of incubation with either 20 or 50 *μ*M silibinin. It was evidenced that silibinin did not induce apoptosis under the experimental conditions of the present study (supplemental Figure 1).

### 3.2. Effect of Silibinin on Angiotensin II-Induced Cardiomyogenesis and Contraction Frequency

**Figure 2:**
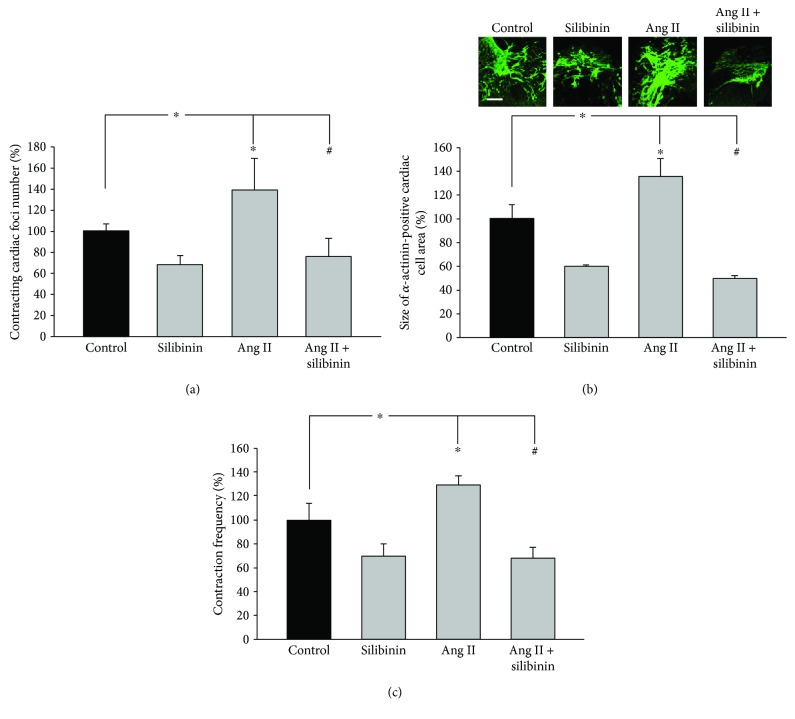
Inhibitory effect of silibinin on Ang II-induced cardiomyogenesis and contraction frequency. (a) Number of contracting cardiac foci differentiated from ES cells (*n* = 5), (b) size of cardiac cell areas (*n* = 4), and (c) contraction frequency (*n* = 5). The images in (b) show representative cardiac cell areas immunolabeled with an antibody against *α*-actinin. The bar represents 300 *μ*m. Embryoid bodies were treated from day 3 to day 14 of cell culture with either silibinin (20 *μ*M), Ang II (1 *μ*M), or a combination of both. ^∗^ *P* ≤ 0.05, significantly different to the untreated control. ^#^ *P* ≤ 0.05, significantly different to the Ang II-treated sample.

Previous studies have shown that Ang II stimulated the cardiomyogenesis of ES cells [17]. Moreover, a recent study demonstrated that silibinin may act as an Ang II receptor 1 (AT_1_) antagonist [9]. Since the data of the present study evidenced that silibinin decreased the cardiomyogenesis of ES cells and the frequency of contractions, we investigated whether silibinin would interfere with Ang II-induced cardiomyogenesis and contraction frequency. To characterize the effect of silibinin on Ang II-mediated cardiomyogenesis of ES cells, the number of contracting cardiac foci was counted from day 7 to day 14 of the cell culture either in the absence or presence of silibinin. It was apparent that Ang II (1 *μ*M) increased the number of cardiac foci, which was completely blunted upon coincubation with silibinin (20 *μ*M) (Figure 2(a)). Moreover, we assessed the size of *α*-actinin-positive cell areas on day 14 and demonstrated that Ang II significantly increased cardiac cell areas, which was completely abolished in the presence of silibinin (Figure 2(b)). To investigate whether the contraction frequency was affected by silibinin and Ang II treatment, we calculated the frequency of contractions per minute. We found that the contraction frequency was significantly increased upon Ang II treatment compared to the untreated control, whereas silibinin (20 *μ*M) alone significantly decreased contraction frequency. Preincubation with silibinin abolished the increase in contraction frequency achieved with Ang II (Figure 2(c)).

### 3.3. Effect of Silibinin on Ca^2+^ Oscillations upon Angiotensin II Treatment

**Figure 3:**
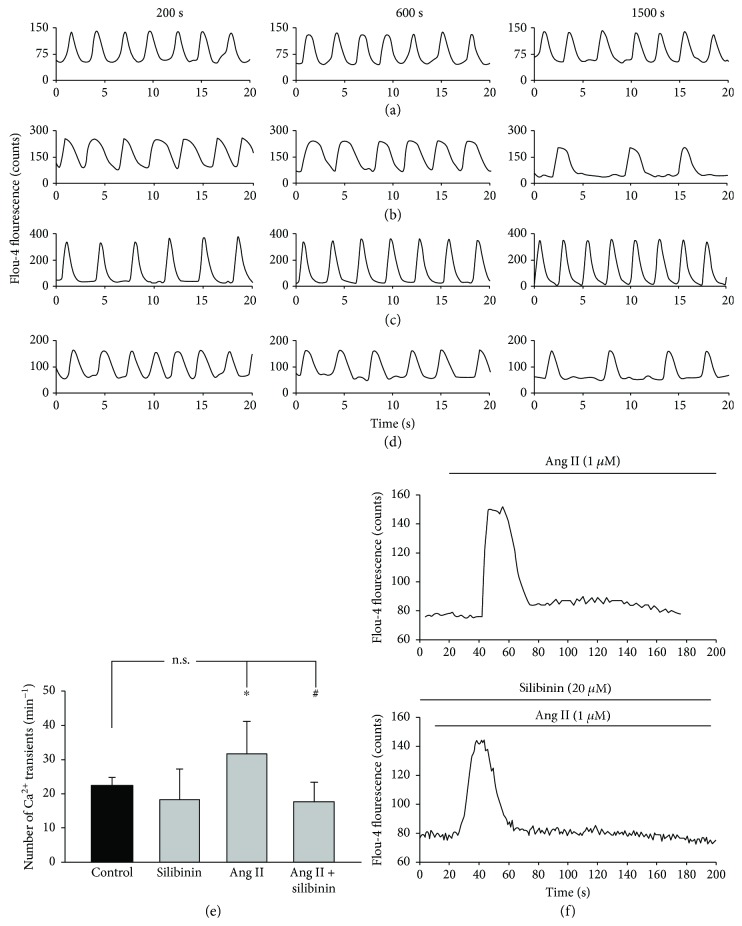
Effects of Ang II and silibinin on the frequency of Ca^2+^ transients in cardiac cells differentiated from ES cells. Cardiac cells were enzymatically dissociated from 7-day-old embryoid bodies and labeled on day 8 with the Ca^2+^-sensitive fluorescence dye Fluo-4. Ca^2+^ spiking was evaluated in 3 different time windows, i.e., 200 s, 600 s, and 1500 s. Shown are representative traces of individual cells. (a) Untreated controls, (b) silibinin- (20 *μ*M) treated cells, (c) Ang II- (1 *μ*M) treated cells, and (d) cells treated with a combination of Ang II (1 *μ*M) and silibinin (20 *μ*M). The bar chart in (e) shows the means ± S.D. of 10 experiments. ^∗^ *P* ≤ 0.05, significantly different to the untreated control. ^#^ *P* ≤ 0.05, significantly different to the Ang II-treated sample. (f) Representative Ca^2+^ transients in rat smooth muscle cells. Upper panel: cells were treated with Ang II (1 *μ*M) and changes in Fluo-4 fluorescence were recorded. Bottom panel: cells were preincubated for 60 min with silibinin (20 *μ*M) and subsequently treated with Ang II (1 *μ*M) (*n* = 3).

Spontaneous contractions and action potentials in cardiac cells are associated to rhythmic Ca^2+^ oscillations. Since our data demonstrated that Ang II treatment stimulated the cardiomyogenesis of ES cells, we investigated whether Ang II treatment would have an impact on cardiac cell function. To achieve this aim, contracting embryoid bodies (day 7 of cell culture) were enzymatically dissociated, labeled with the Ca^2+^-sensitive fluorescence dye Fluo-4, AM on day 8, and intracellular Ca^2+^ oscillations were recorded in single cardiac cells after different times of incubation (200 s, 600 s, and 1500 s) with either Ang II (1 *μ*M), silibinin (20 *μ*M), or a combination of both. It was evident that silibinin treatment decreased the frequency of Ca^2+^ spikes (Figures 3(b) and 3(e)) as compared to the untreated control (Figures 3(a) and 3(e)). In contrast, an increase in spiking frequency was observed upon Ang II treatment (Figures 3(c) and 3(e)). However, when Ang II was applied with silibinin, the stimulation of Ca^2+^ spiking frequency was abolished, which indicates that silibinin interferes with Ang II-mediated signaling pathways (Figures 3(d) and 3(e)).

### 3.4. Effect of Silibinin on Ang II-Induced Ca^2+^ Responses in Rat Smooth Muscle Cells

A previous report on CHO cells overexpressing the AT_1_ receptor suggested that silibinin may be an AT_1_ receptor antagonist that inhibited the Ang II-mediated Ca^2+^ response [9]. To examine this assumption, rat smooth muscle cells, which are well known to express the AT_1_ receptor [21], were exposed to Ang II (1 *μ*M) in the absence or presence of silibinin (20 *μ*M) (Figure 3(f)). It was observed that Ang II raised Ca^2+^ even in the presence of silibinin, which suggests that silibinin does not affect AT_1_ receptor function, but may interfere with Ang II-mediated signaling pathways downstream of the AT_1_ receptor. The Ang II-induced Ca^2+^ response could not be inhibited at silibinin concentrations up to 100 *μ*M (supplemental Figure 2).

### 3.5. Silibinin Inhibits Ang II-Mediated Activation of ERK1/2, JNK, and p38

**Figure 4:**
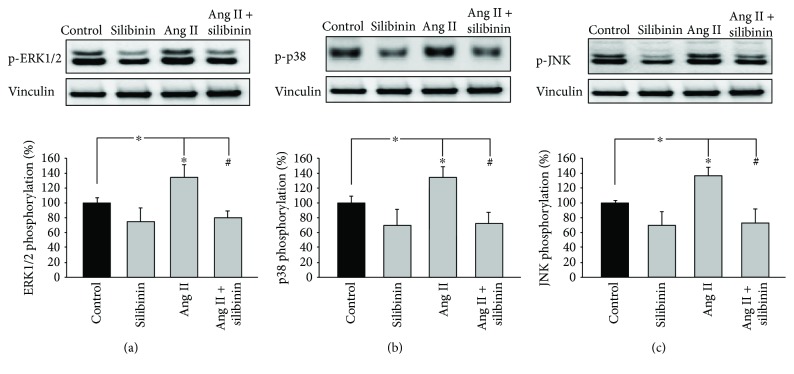
Effects of Ang II and silibinin on the activation of (a) ERK1/2 (*n* = 6), (b) p38 (*n* = 5), and (c) JNK (*n* = 5). 6-Day-old embryoid bodies remained either untreated or were treated with Ang II (1 *μ*M), silibinin (20 *μ*M), or a combination of both. MAPK activation was monitored after 15 min of incubation with Ang II by western blot analysis using phosphospecific antibodies. Shown are representative western blots. The bar charts show the means ± S.D. of (*n* = 6) experiments for ERK1/2 and (*n* = 5) experiments for p38 and JNK, respectively. ^∗^ *P* ≤ 0.05, significantly different to the untreated control. ^#^ *P* ≤ 0.05, significantly different to the Ang II-treated sample.

Since silibinin did not affect the Ang II-mediated Ca^2+^ response, we assumed that it may interfere with downstream signaling cascades. Since it has been previously shown that Ang II activates ERK1/2, JNK, and p38 in differentiating ES cells [17], we investigated whether silibinin (20 *μ*M) would abolish MAPK activation upon treatment of embryoid bodies with Ang II (1 *μ*M). Indeed, silibinin treatment of 6-day-old embryoid bodies efficiently abolished the Ang II-mediated activation of ERK1/2 (Figure 4(a)), p38 (Figure 4(b)), and JNK (Figure 4(c)) as evaluated using phosphospecific antibodies. These data corroborated our assumption that silibinin interfered with Ang II signaling downstream of the AT_1_ receptor.

### 3.6. Effect of Silibinin on FGF-2 and EPA-Induced Cardiomyogenesis of ES Cells

**Figure 5:**
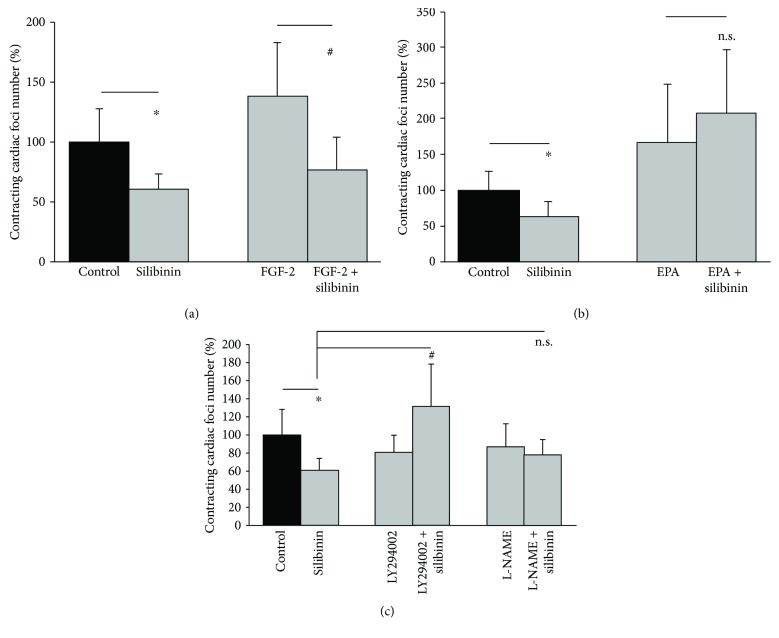
Effect of the procardiogenic agents FGF-2 (a) and EPA (b) on the inhibition of cardiomyogenesis by silibinin. Embryoid bodies were treated from day 3 to day 10 of differentiation with silibinin (20 *μ*M) alone or with either FGF-2 (10 ng/ml) (*n* = 5) or EPA (50 *μ*M) (*n* = 5) in the absence or presence of silibinin. On day 10, the number of contracting cardiac foci was counted. (c) Interference with PI3-K and NO signaling and its impact on the inhibition of cardiomyogenesis by silibinin. Embryoid bodies were treated from day 3 to day 10 with either silibinin (20 *μ*M) alone or with either the PI3-K inhibitor LY294002 (5 *μ*M) (*n* = 5) or L-NAME (100 *μ*M) (*n* = 5) and the number of contracting cardiac foci was counted. ^∗^ *P* ≤ 0.05, significantly different to the untreated control. ^#^ *P* ≤ 0.05, significantly different to the FGF-2 or LY294002-treated sample. n.s., not significant.

Treatment of differentiating ES cells with either FGF-2 [22] or the omega-3 polyunsaturated fatty acid EPA [23] has been previously demonstrated to stimulate cardiomyogenesis. To investigate whether silibinin would block the stimulatory effect of other agents on the cardiomyogenesis of ES cells, embryoid bodies were treated from day 3 to day 10 of differentiation with either FGF-2 (10 ng/ml) or EPA (50 *μ*M) in the absence or presence of silibinin (20 *μ*M), and the number of spontaneously contracting cardiac foci was assessed (Figures 5(a) and 5(b)). Our data demonstrated that silibinin completely inhibited the stimulation of cardiomyogenesis by FGF-2 (Figure 5(a)). In contrast, the stimulation of cardiomyogenesis by EPA could not be blocked upon coincubation with silibinin (Figure 5(b)). In further experiments, we investigated whether interference with signaling pathways, i.e., phosphoinositide 3-kinase PI3-K or nitric oxide (NO) which have been previously shown to be important in silibinin-driven cellular changes [24], would block the inhibitory effect of silibinin on cardiomyogenesis. The results of these experiments (Figure 5(c)) showed, that indeed inhibition of PI3-K by LY294002 (5 *μ*M) abolished the anticardiomyogenic effect of silibinin and even stimulated cardiomyogenesis above the level of the untreated control. Moreover, inhibition of endothelial NO synthase (eNOS) partially abolished the adverse action of silibinin on the cardiomyogenesis of ES cells.

## 4. Discussion

Previous studies have shown that the vasoactive hormone Ang II stimulated the cardiomyogenesis [17] as well as smooth muscle cell differentiation [18] of ES cells. Moreover, a recent study evidenced that silibinin may act as an AT_1_ receptor antagonist [9].

The data of the present study demonstrated that silibinin dose-dependently inhibited cardiomyogenesis of ES cells. Moreover, silibinin decelerated the frequency of Ca^2+^ spikes in differentiated cardiac cells. To investigate whether the effects of silibinin on cardiomyogenesis and cardiac cell function were due to the inhibition of Ang II-mediated signaling pathways, we investigated whether silibinin treatment would abolish the stimulation of cardiomyogenesis achieved upon Ang II treatment of differentiating ES cells. In corroboration with the data of Wu et al. [17], we observed the stimulation of cardiomyogenesis following incubation with Ang II. Moreover, Ang II treatment increased the contraction frequency of cardiac areas differentiated from ES cells and the frequency of Ca^2+^ spikes in differentiated cardiac cells. The stimulation of cardiomyogenesis as well as the increase in Ca^2+^ spiking frequency achieved with Ang II was completely abolished upon cotreatment with silibinin, supporting the notion that silibinin is interfering with Ang II signaling. Previous studies of Wu et al. [17] and Zheng et al. [18] suggested that the effects of Ang II on cardiac and smooth muscle cell differentiation were mediated via the AT_1_ receptor, since the specific AT_1_ receptor antagonist losartan abolished the observed effects. Notably, silibinin has been discussed to act as an AT_1_ receptor antagonist in CHO cells which were stably transfected with the human AT_1_ receptor [9]. We therefore investigated whether silibinin would abolish the Ang II-mediated Ca^2+^ response in smooth muscle cells which are well known to express the AT_1_ receptor [25]. Interestingly, it was observed that silibinin was not able to inhibit the Ca^2+^ response elicited by Ang II even at high (100 *μ*M) concentrations. Thus, the data of the present study argue against an involvement of AT_1_ receptor inhibition by silibinin at least in the physiological Ang II concentrations (1 *μ*M) used in our experiments.

If the AT_1_ receptor activity and Ca^2+^ signaling are not affected, silibinin could possibly interfere with the signaling cascade further downstream. It has been previously described in rat neonatal cardiomyocytes that Ang II activates ERK1/2, p38, and JNK, whereby the phosphorylation of p38 and JNK is dependent on reactive oxygen species (ROS) generation [26]. In the experiments of the present study, silibinin significantly inhibited ERK1/2, p38, and JNK activity as compared to the untreated control, whereas MAPK stimulation was observed upon Ang II treatment. According to our assumptions, silibinin totally abolished the stimulation of all members of the MAPK family by Ang II, which indicates that the compound interferes with the MAPK signaling cascade downstream of the AT_1_ receptor. Moreover, silibinin blunted the procardiomyogenic effect of FGF-2, which should be expected since FGF-2 signaling has several crossovers with Ang II signaling, including the activation of MAPK pathways. In contrast, silibinin failed to abolish the EPA-induced stimulation of cardiomyogenesis, suggesting that EPA activates signaling pathways which are distinct from Ang II signaling and MAPK activation. Recent data from our group [24] demonstrated that silibinin increases nitric oxide (NO) generation and activates endothelial NO synthase. Notably, inhibition of NO generation by L-NAME partially reversed the adverse effect of silibinin on cardiomyogenesis. Moreover, inhibition of PI3-K reversed the silibinin effect and even stimulated cardiomyogenesis above the level of the untreated control. PI3-K is well known to activate eNOS which generates NO [27]. Since NO is well known as a free radical scavenger for ROS [28], it may be assumed that the inhibitory effect of silibinin on MAPK activity may be due to its capacity to raise NO concentration in the tissue. Previous data from others who showed that silibinin protects H9c2 cardiac cells from oxidative stress and inhibits phenylephrine-induced hypertrophy, presumably by repression of the phenylephrine-induced phosphorylation of ERK1/2 kinases [29], are pointing to the same direction. Moreover, the property of silibinin to act as a free radical scavenger has been validated in several studies [24, 30, 31].

The RAAS has been shown to exert a deep impact on cardiac development [32]. In humans, all components of RAAS are expressed at very early stages of embryogenesis (30–35 days of gestation) in different organs, suggesting that Ang II likely plays a role in the growth and differentiation of various organotypic cells [33]. Although triple knockouts of the AT_1a_, AT_1b_, and AT_2_ receptors are viable and fertile, the lack of both AT_1_ subtypes was associated with atrophic changes in the myocardium, a reduced coronary flow, and a reduced left ventricular systolic pressure [34, 35]. Recently, it has been outlined that antihypertensive medication of pregnant women is associated with an increased risk for congenital heart defects. This was the case for the treatment with *β*-blockers as well as with the use of renin-angiotensin system blockers [36]. Milk thistle seeds as well as their pharmacologically active ingredients are frequently used as dietary herbal supplements mainly to detoxify the liver. Since the data of the present study demonstrate that silibinin inhibits cardiac differentiation of ES cells and affects Ang II-mediated signaling cascades, its use should be avoided in pregnant women.

## Data Availability

There are no data in the present study which have been published previously.

## Abbreviations

Ang II: Angiotensin II
AT_1_: Angiotensin II type 1
Ca^2+^: Calcium
CHO: Chinese hamster ovary
DOCA: Deoxycorticosterone acetate
ECL: Enhanced chemiluminescence
eNOS: Endothelial NO synthase
EPA: Eicosapentanoic acid
ERK1/2: Extracellular signal-regulated kinase 1/2
FCS: Foetal calf serum
FGF-2: Fibroblast growth factor-2
HRP: Horseradish peroxidase
JNK: C-Jun NH_2_-terminal kinase
IGF: Insulin-like growth factor
MAPK: Mitogen-activated protein kinase
NO: Nitric oxide
PBS: Phosphate-buffered saline
RAAS: Renin-angiotensin aldosteron system
ROS: Reactive oxygen species.

## Supplementary Materials

Supplementary Materials Supplemental Figure 1: effect of silibinin on apoptosis induction in differentiating ES cells. Embryoid bodies were treated from day 3 to 10 of differentiation with silibinin (20, 50 *μ*M) and cleaved caspase 3 expression was evaluated by western blot (*n* = 5). *β*-Actin was used as loading control. n.s., not significant. Supplemental Figure 2: effect of preincubation (60 min) with silibinin (100 *μ*M) on Ang II-induced Ca^2+^ transients in adult rat smooth muscle cells.
