# Supplementary material for: The Milk Thistle (Silybum marianum) Compound Silibinin Inhibits Cardiomyogenesis of Embryonic Stem Cells by Interfering with Angiotensin II Signaling
Source: Stem Cells Int. 2018 Dec 13;2018:9215792. doi: 10.1155/2018/9215792 (PMC6311720; doi:10.1155/2018/9215792)
Supplement: Supplementary Materials — Supplemental Figure 1: effect of silibinin on apoptosis induction in differentiating ES cells. Embryoid bodies were treated from day 3 to 10 of differentiation with silibinin (20, 50 μM) and cleaved caspase 3 expression was evaluated by western blot (n = 5). β-Actin was used as loading control. n.s., not significant. Supplemental Figure 2: effect of preincubation (60 min) with silibinin (100 μM) on Ang II-induced Ca2+ transients in adult rat smooth muscle cells. [file 9215792.f1.pdf]

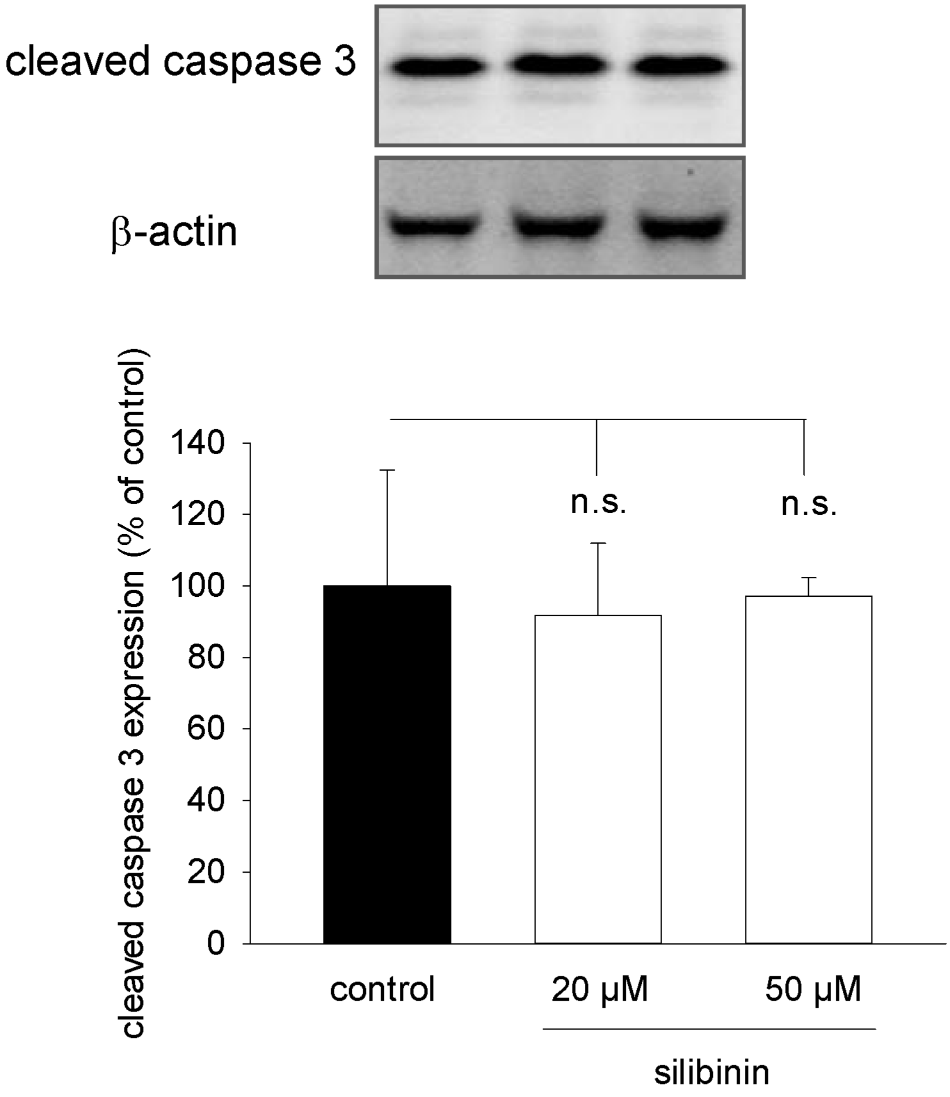

Supplemental Fig. 1. Effect of silibinin on apoptosis induction in differentiating ES cells. Embryoid bodies were treated from day 3 to day 10 of differentiation with silibinin (20, 50 µM) and cleaved caspase 3 expression was evaluated by western blot (n = 5). ß-actin was used as loading control. n.s., not significant.

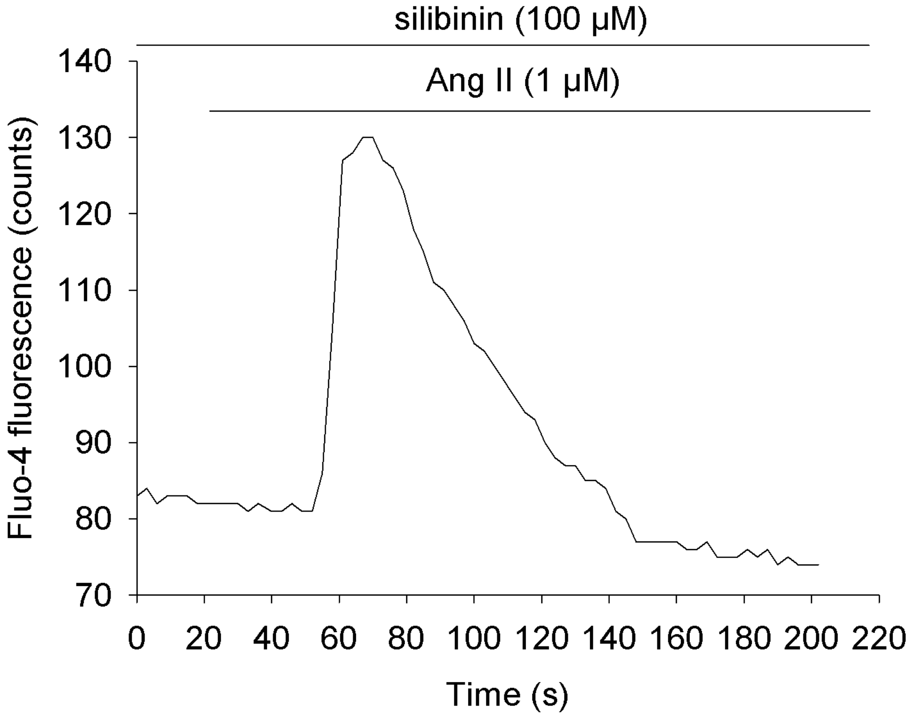

Supplemental Fig.2. Effect of pre-incubation (60 min) with silibinin (100 µM) on Ang II-induced Ca^{2+} transients in adult rat smooth muscle cells.
